# Smartphone-Based Health Program for Improving Physical Activity and Tackling Obesity for Young Adults: A Systematic Review and Meta-Analysis

**DOI:** 10.3390/ijerph17010015

**Published:** 2019-12-18

**Authors:** Han-Na Kim, Kyoungsan Seo

**Affiliations:** 1 Department of Dental Hygiene, College of Health and Medical Sciences, Cheongju University, Cheongju 28503, Korea; hnkim@cju.ac.kr; 2 Department of Nursing, College of Health and Medical Sciences, Cheongju University, Cheongju 28503, Korea

**Keywords:** behavior change, mobile phone, young adult, physical activity, meta-analysis

## Abstract

The need for physical activity for health promotion is recognized, yet young adults still perform insufficient physical activity. Smartphone health programs can be applied easily without time and space constraints, and various mobile health programs based on smartphone applications have recently been developed and applied. This study aimed to measure the effects of mobile smartphone-based health programs on physical activity and obesity outcomes in young adults through a systematic review and meta-analysis. We searched publications in English through electronic databases up to May 2019. Studies were included that provided interventions to improve physical activity using smartphone applications for young adults. After assessing study quality, data were extracted and synthesized concerning whether smartphone interventions affect health outcomes including physical activity and weight using Meta-Analysis software. Four randomized controlled studies and a quasi-experimental study were analyzed. They provided information related to health management, diet, physical activity, and personalized feedback using smartphone applications. The meta-analysis showed that smartphone-based health interventions significantly affect weight loss and increase physical activity. This study provides modest evidence for using smartphone health programs to improve young adults’ physical activity, weight control, and body mass index (BMI). Future research is needed to understand long-term effects and the reliability of increasing physical activity through smartphone health programs.

## 1. Introduction

In many industrialized nations, the vast majority of adults do not meet the physical activity guidelines of 150 min per week [1]. Physical inactivity is now identified as the fourth leading risk factor for obesity and global mortality. Physical inactivity levels are rising in many countries with major implications for the prevalence of non-communicable diseases (NCDs) and the general health of the population worldwide [2]. The amount of physical activity of young adults is still below the amount recommended by the World Health Organization. The sedentary lifestyle of these young adults will be the main cause of various future health problems. Physical activity is an important primary and secondary prevention strategy for non-communicable conditions, and is considered a challenge for healthcare providers in a variety of environments and in academia [3].

Physical activity associated with work, home, and transportation has decreased because of technological advancements and social changes. On the other hand, young adults, who conduct many of their daily activities using smartphones and have been called “phono-sapiens”, have a desire to take time out for themselves and to make health management fun [4]. Mobile phones and smartphone applications are a special means of communication that may be used to deliver health services and health information [5]. Physical activity promotion programs have been proposed that use this technology [6].

There are various factors that can change health behaviors, such as the environment, social policies, and internal motivational changes. The behavioral change of increased physical activity may also affect other health behaviors, such as dietary habits, weight control, and balanced nutrition intake. This health behavior change was argued to ultimately lead to weight loss and disease prevention [7]. Therefore, school- and workplace-based health promotion programs, which have been recently introduced and use mobile devices, have introduced cases in which intervention programs for improving physical activities provide integrated and personalized interventions for the improvement of physical activity, obesity, and nutrition [8,9,10]. In these intervention programs, changes in the amount of physical activity and body weight and the improvement of nutrition status have often been identified as results.

Mobile health care services refer to healthcare services with which people can manage health anytime and anywhere by measuring body information related to health, even on the go. It is provided by a wearable or portable sensor that can periodically and stably measure body information and a device that receives this information [11]. In the case of smartphone-based health intervention, it can be seen as a form that fuses the functions of mobile health care and various app functions of smartphones. To date, systematic literature reviews and meta-studies on physical activity have identified whether it contributed to health promotion [12] or reported the effect on the promotion of physical activity of patients with diseases such as human immunodeficiency virus (HIV) [13], cardiovascular disease, or diabetes [14] by using wearable equipment and collecting bio-data. In addition, a mobile health intervention for adolescents under the age of 18 years and their caregivers was reported to have a significant effect on changes in health behavior or disease control [10], and a meta-study verified the effect of a physical activity mobile intervention, regardless of the age of the subjects [15]. However, there have not been many studies solely on young adults, and the results have varied from study to study [8,9,10,11]. Furthermore, there has been no meta-analytic study. Mobile health interventions and personal digital assistants (PDA) and Internet-based interventions have been conflated and analyzed together. Given the difference between mobile health and smartphone-based interventions, it is necessary to identify whether intervention strategies using smartphones can influence health results such as physical activity and obesity of young adults and explore the intervention strategies further.

Accordingly, this study intended to identify the effects of smartphone-based health intervention programs provided for young adults on health outcomes, including physical activity promotion, and the essential factors of the interventions through a systematic literature review and meta-analysis research method.

## 2. Methods

This study consisted of a systematic literature review and meta-analysis to analyze the contents of smartphone-based physical activity promotion programs for young adults and evaluate their effects. The meta-analysis was conducted in accordance with the preferred reporting items for systematic reviews and meta-analyses (PRISMA 2015) guidelines [16].

### 2.1. Search Strategy

This study used specialized search sites to find studies for the analysis. The authors searched using medical subject headings (MeSH) terms in Medline, cumulative index to nursing and allied health (CINAHL), the Cochrane Library, open dissertations, and academic searches. The terms confirmed through the preliminary investigation were mobile health OR smartphone OR mobile apps OR mobile application OR apps OR application AND physical activity OR exercise AND young adult. The authors searched under the condition of including all the academic articles published by the search date (May 2019) by using the search words on each search site, when full text search was available. The researchers aimed to increase the sensitivity of the data search by additionally conducting a manual search after the above search. The researchers collected a list of data extracted from each site in a bibliography program and first excluded duplicated articles. Then, the process of selecting papers for the analysis was conducted, based on the selection and exclusion criteria. All processes were conducted separately by two researchers, and if they were inconsistent, they discussed them several times until they became consistent and selected the final analysis subjects.

### 2.2. Inclusion Criteria

The criteria for selecting studies were that they used smartphone-based apps as the primary intervention, included physical activity, body weight, or body mass index (BMI) in the result measurement indicators, and that they were conducted on healthy young adults aged 19 to 35 years. In addition, studies using randomized trials and quasi-experimental designs with comparison groups were included.

### 2.3. Risk of Bias Appraisal

The tool used for evaluating the quality of the articles was Risk of Bias [17]. This study used the functions provided by Comprehensive Meta-Analysis, version 2.0 (Biostat, Englewood, NJ, USA). Two evaluators carried out an evaluation of the quality of the articles independently after reading the original texts. Then, when there was a difference in the scores between the evaluators, an agreed score was determined through discussion.

### 2.4. Data Extraction and Synthesis

For the subject studies that went through the data selection process, this study extracted the contents in accordance with the framework of PICOs; population, intervention, comparison, outcomes and study design. The main contents were the characteristics of the subjects (number of people and averaged age), intervention programs provided (content composition, time, number of interventions, providers, etc.), programs provided for comparison groups, outcomes (indicators, valid results, and time of measurement), and study designs. In addition, this study extracted the authors’ names, publication year, behavioral change theories applied, measurement methods for physical activity, and strategies used for applying the interventions. The researchers analyzed physical activity, body weight, and BMI, which were the outcome variables in each study, in the meta-analysis.

### 2.5. Statistical Analysis

For the meta-analysis, this study used a statistical software program, Comprehensive Meta-Analysis, version 2.0 (Biostat, Englewood, NJ, USA). Results were considered statistically significant when the *p* value < 0.05 in a two-tailed test. In the selected papers, this study analyzed the effect size for changes in physical activity, BMI, and body weight, and tested the statistical homogeneity. To assess heterogeneity qualitatively, we used the I-squared (I^2^) statistic after forest plots were manually inspected and compared study characteristics quantitatively [17]. Mean difference (MD) was used when the same scale was used in all studies, and standardized mean difference (SMD) was employed when different scales were used [18]. The standard deviation (SD) was calculated by employing the *p*-value and mean for the meta-analysis for the paper [19], which presented the SD in 25–75% quartiles.

## 3. Results

### 3.1. Search Outcomes

**Figure 1:**
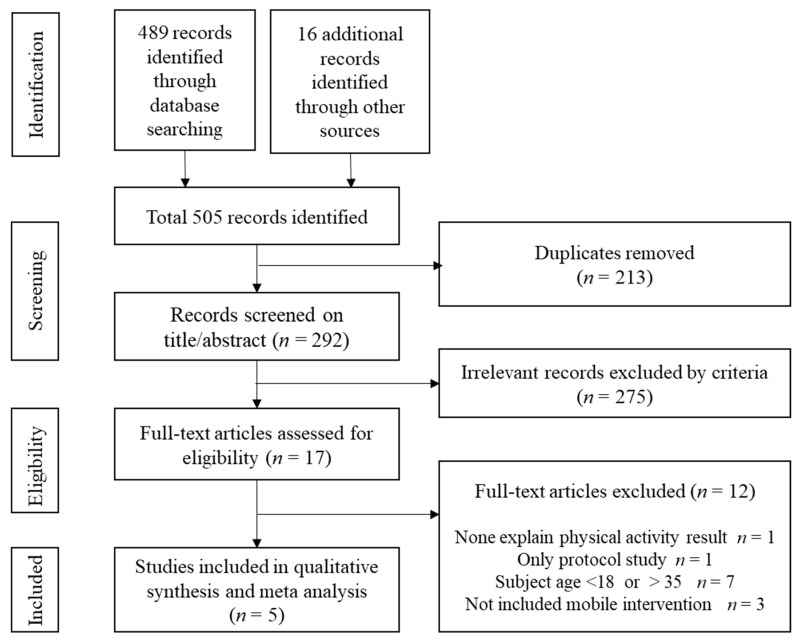
Flow of the preferred reporting items for systematic reviews and meta-analyses (PRISMA).

In total, 505 records were identified. After removal of duplicates, titles and abstracts were read, and a further 275 articles were removed based on the selection criteria. Eleven full text articles were assessed. Several articles were excluded because they did not measure the amount of physical activity, were introduced as protocols of interventional studies, the subjects were not young adults, or they did not include a mobile intervention. Five met the criteria for inclusion and were included in the meta-ethnography (Figure 1).

### 3.2. Characteristics of Included Studies

**Figure 2:**
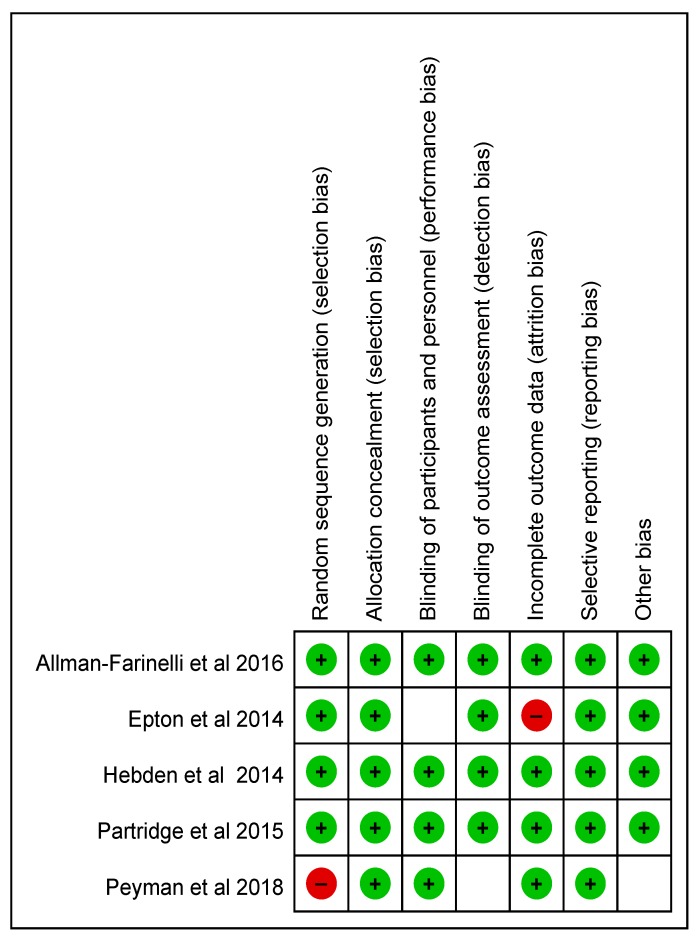
Risk of bias. Note: green circle, low risk of bias; red circle, high risk of bias; empty box, unclear risk of bias.

Five papers [19,20,21,22,23] were included in the analysis. In assessing the quality of research through the risk of bias, four papers with a randomized controlled trial (RCT) research design were evaluated and one with a quasi-experimental design (Figure 2). The publication years were from 2006 to 2019. The theories of health behavior change used in the program development were the transtheoretical model (TTM) and the Theory of Planned Behavior (TPB). TTM explains that behavioral change can be divided into stages of change and behavioral changes centering on internal motivational changes [24]. TPB is also used to improve health behaviors [25]. The main tool used to measure the amount of physical activity was the International Physical Activity Questionnaire (IPAQ), in which the amount of physical activity over the past seven days was recalled and answered in the questionnaire. Not all studies reported results by intensity of physical activity. Therefore, the total amount of physical activity was identified as an outcome. The studies included were diverse and details of each are included in Table 1 and Table 2.

### 3.3. Data Extraction from Systematic Review

#### 3.3.1. Participants

A total of 1830 people were included in the meta-analysis. Recruitment of the research subjects was conducted in the universities where the studies were carried out or through local community advertisements. Two studies [21,23] recruited subjects who could use the mobile health program for obesity prevention or weight loss with a mobile phone and had a BMI of at least 23 kg/m^2^, weight gain of at least 2 kg in the previous 12 months, or a BMI of 25.0 kg/m^2^ to 31.9 kg/m^2^. Most of the studies were carried out on adults who wanted to voluntarily participate in the programs. The number of subjects ranged from 51 to 1107, and dropout rates ranged from 0% to 25.4% (Table 1).

#### 3.3.2. Program Contents Provided to Intervention and Control Groups

The intervention programs consisted of lifestyle intervention, education and coaching to promote physical activity, dietary counseling, education related to health behavior, and feedback which were provided 5–24 times over two to six months. By using smartphone applications as an intervention strategy, the subjects could directly record their health and nutrition status and receive feedback. Text messages or emails tailored by the information a subject provided were sent, or education and coaching calls personalized depending upon their health and nutrition status were offered. In some cases, smartphone apps and additional web-based education were provided, and devices were offered to check lecture-style education and steps walked. In the study that provided a step count device, the comparison group was also offered the device but did not give feedback on the results of their steps.

Dietitians and health care center staff functioned as intervention providers, and some studies did not identify the intervention providers. Follow-ups were conducted one week to nine months after the end of the studies. Two studies did not provide any intervention for the comparison groups; two studies contacted participants via text message for education or brief guidance after the study was finished; and another provided the intervention after the program was completed.

#### 3.3.3. Outcomes

Five studies were conducted aimed at preventing obesity and losing body weight by promoting physical activity and improving diet. They identified the results of physical activity (mean score of knowledge, attitude and level of physical activity, IPAQ, daily step counts, and muscular fitness), physiological indicators (weight, BMI, etc.), and health behaviors (diet behavior changes; categories of intake for fruits, vegetables, sugar-sweetened beverages (SSBs) and take-out meals, daily intake of fruit and vegetables). Significant differences in results between the intervention and control groups were found for physical activity (one study), diet behavior change (two studies), smoking rates (one study), and self-reported weight (one study). However, in some cases, the effect differed by measurement indicators (Table 1).

### 3.4. Meta-Analysis of the Results

**Figure 3:**
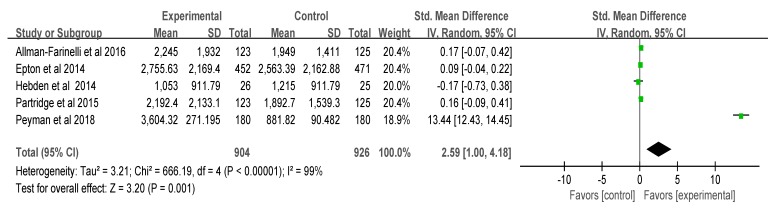
Effects on physical activity. Note: green color, random effect and confidence interval in each study; black, total effect.

**Figure 4:**
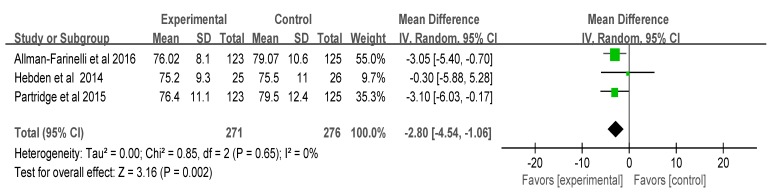
Effects on body weight. Note: green color, random effect and confidence interval in each study; black, total effect.

**Figure 5:**
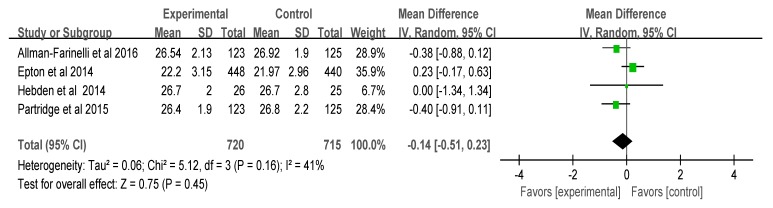
Effects on body mass index. Note: green color, random effect and confidence interval in each study; black, total effect.

The standard mean difference (SMD) of the five papers (*n* = 1830) on the change in physical activity was 2.59 (I^2^ = 99%, *p* = 0.001, confidence interval (CI): 1.00, 4.18), indicating a significant increase in physical activity in the intervention groups. The combined mean difference of the three papers on weight change was −2.80 (I^2^ = 0%, *p* = 0.002, CI: −4.54, −1.06), which suggested significant weight loss in the intervention groups. However, the combined mean difference of the four papers on BMI change was −0.14 (I^2^ = 41%, *p* = 0.45, CI: −0.51, 0.23), and a significant difference was not found between the two groups (Figure 3, Figure 4 and Figure 5). That is, the smartphone-based intervention programs had positive effects on increased physical activity and on weight loss, but effects on BMI were not found.

## 4. Discussion

Advances in smart devices and Internet technologies have led to the utilization of mobile health programs, and health intervention programs for adults have been conducted to lose body weight, promote health, and improve dietary habits. This study identified the effects on physical health promotion and weight loss of young adults in studies using mobile health programs through a systematic literature review and meta-analysis. The intervention programs consisted of lifestyle interventions, education and coaching to promote physical activity, dietary counseling, and health behavior-related education. Therefore, individuals’ behavioral changes were recorded, personalized coaching and education were conducted, and feedback was given via text message, email, phone calls, or mobile health devices. Mobile health was found to be effective for adults who had difficulty visiting health centers or meetings because of increasing obesity. In addition, as the programs can be made available in a wide range of regions at a low cost, the programs are effective for young adults who use smart devices.

The meta-analysis in this study identified increases in physical activity. Another meta-study on middle-aged adults showed significant results for changes in physical activity and steps. The changes in physical activity among young adults in this study seemed to be statistically significant, but the heterogeneity (I^2^) of the five papers was large. The reason for this might be that Peyman’s study had a large deviation and relatively large increase in physical activity compared to the other four studies, and also because it used a quasi-experimental design. Variability in the participants, interventions, and outcomes studied may be considered as clinical heterogeneity, and variability in study design, outcome measurement tools, and risk of bias may be described as methodological heterogeneity. Variability in the intervention effects being evaluated in the different studies is known as statistical heterogeneity, and is a consequence of clinical or methodological diversity, or both, among the studies [26].

In the meta-analysis of Direito et al. [27], a consistent result could be found, since mHealth intervention was found to have a small effect (SMD: 0.14) on physical activity. The research of Carter et al. [28] on mobile health programs for promoting physical activity reported that personal factors and features of the device influenced the experience of using mobile health to support physical activity. It also reported that the programs functioned positively for the thoughts, perceptions, strategies, and motivation to promote physical activity. According to the results of the review paper by Stuckey et al., [29], eight out of 18 studies applying smartphone-based interventions showed an increase in physical activity or steps. For young adults, we believed that as smartphone apps are able to always be with them, they could be effective behavior change support systems. Because the research subjects of the paper were adults, including elderly people, it was different from this study in terms of research subjects and intervention methods, and mainly identified step counts as the outcome variable. Young adulthood is a time when social life is so busy that one does not have enough time to take care of their health and also disease begins to develop. In other words, it also can be a time to prevent disease. Low physical activity and obesity are the leading causes of many diseases. According to Allman-Farinelli et al. [20], providing multi-component intervention through a smartphone app has been effective for both physical activity and weight loss, and has maintained long-term effectiveness. Compared to the face-to-face program offered for a certain period, the smartphone apps can be used at a low cost and used as long as the subject or manager wanted, so it would be expected to have a long-term effect. In addition, tailored feedback and information at that time were important strategies, and the subjects could directly record their health and nutrition status through the application [11]. Subjects who recorded their own behavior could highlight their concerns about their health and evaluate themselves. We discussed real time interaction as a function of smartphone apps. In particular, for healthy young adults, making goals more specific is important. In the motivational aspects, three studies [19,20,23] were developing their programs based on the transtheoretical model (TTM). TTM is used to change motivation before a behavior change. Before performing physical activity, TTM can help explain and measure the process of change and the preparation of subjects [24].

Compared to the results that mobile health interventions had an effect on changes in body weight in the five studies, one study [19] did not find a statistically significant effect on the change in physical activity (IPAQ). This might be because body weight can be measured more objectively and is a change indicator with a certain direction, while the amount of physical activity, which was measured by recalling the last seven days and answering the questionnaire, might vary depending on the respondent’s subjectivity, recall ability, or severity. In addition, if the subject’s goal is to improve the quality of physical activity, they will not care about the amount of physical activity in the program. For this reason, there was no difference in the amount of physical activity measured by the IPAQ. The International Physical Activity Questionnaire (IPAQ) was used as a standardized measure to estimate habitual practice of physical activities of populations [30]. However, physical activity levels were overestimated at low activity levels and underestimated at high activity levels when compared to heart rate-derived measurements [31]. A more direct method of measuring the amount of physical activity is recommended in future studies, and it will be meaningful to explore changes in attitudes and content regarding physical activity, not only the overall amount. Furthermore, decreases in body weight might have occurred in the intervention programs using smartphones because the subjects adjusted their diet after nutritional counseling.

The systematic literature review found that mobile health interventions had an effect on changes in diet behaviors. The indicators were intake of fruits and vegetables, intake of SSBs, number of times eating out, etc. Many young adults aged 18–35 years have life habits involving little physical activity, a large intake of SSBs, and eating out frequently [32]. Changes in dietary habits could be linked to weight loss. A meta-analysis of the results of three studies confirmed weight loss in the intervention groups. This is consistent with the result of a meta-analysis in a prior study [33] on other groups, and is considered to be the effect of the multi-functional nature of interventions based on smartphone apps. Some apps not only provide nutrition databases but also automatically calculate calories consumed and burned, and provide visual displays of personal data [34].

However, a problem has been recently identified where consumers who purchase mobile healthcare products do not use them consistently. More than half of Fitbit purchasers failed to continue to use them (Fitbit S-1 2015). In the case of the Balance Rewards for healthy choices (BRhc) of the US Walgreen company, which is a health management program in mobile app form, consumers using the service for more than 20 weeks accounted for just 10% of the total subscribers [35]. Therefore, in future studies, it will be important to assess the long-term effects and retention rates of the subjects’ physical activities.

### Limitations of This Study

As not many studies were included in the meta-analysis, there are limitations in interpreting the generalizability of the findings of this study. Therefore, it is recommended that meta-studies analyze more papers in the future. Physical activity and outcome measurements (IPAQ) based on participants’ self-reports for many studies were recorded using mobile applications, and the lack of comment on the reliability of the measurement method through mobile apps can be a limitation of the results analysis. In addition, because not all of the studies used the same application, it was difficult to confirm the reliability of the measurement. It may be meaningful to analyze the results of studies that have directly measured physical activity in future studies. One of the limitations of this study is that it did not analyze according to gender differences. This is because there are not enough studies for a meta-analysis, yet, it suggests analyzing with gender perspectives in future research.

## 5. Conclusions

The meta-analysis results for young adults who used smartphone health programs confirmed significant effects on increased physical activity and weight loss. However, meta-analyses including a large number of studies on physical activity need to be conducted in future studies to check if the heterogeneity of physical activity results was as high as in this study. The evidence from the meta-analysis identified smartphone health programs as useful tools for weight loss and increasing physical activity in young adults.

## Figures and Tables

**Table 1:** Systematic review of included studies.

First Author (Published Year)	Study Design	Participants	Sample Size (I, C); Mean Age (Years)	Experimental Condition			Control Intervention	Follow Up	Measured Outcomes (Significant Outcomes)
				Program Context	Application	Session/Duration			
Allman-Farinelli et al. (2016)	Two-arm, parallel, RCT	Overweight over 18–35 year olds with a BMI ≥ 23 kg/m^2^.	248 (I = 123, C = 125); 28.1, 27.2	12 weeks multicomponent diet program and 60 min physical activity; 5 coaching calls with motivational interviewing, personalized text messages, nutrition booklet with physical activity guidelines through web and apps.	Study website and mobile phone app	12 weeks + 2 booster session at 5 months and 8 months	12 weeks, minimal intervention with 4 text messages, 2-page handout based on the dietary guidelines and physical activity guidelines	9 months	Weight (kg), BMI, physical activity (MET-minutes), and diet behaviors (intake of fruits, vegetables, SSBs, and take-out meals)
Hebden et al. (2014)	Two-arm, parallel, RCT	University students and staff	51 (I = 26, C = 25); 22.6, 23.1	Printed diet booklet with instructions. Text messages and email; had access to smartphone applications and Internet forums.	Mobile phone app	12 weeks	Printed diet booklet with instructions	13 weeks	Physical activity (IPAQ), BMI, usual weekly intake of SSB and energy-dense takeaway meals, and daily intake of fruit and vegetables
Epton et al. (2014)	Two arm, RCT	University students	1107 (I = 549, C = 558); 18.7, 19.0	Theory-based persuasive messages were developed to encourage regular exercise and fruit and vegetable intake, and to discourage binge drinking and smoking. Includes a mixture of text and video links to other relevant material.	Smartphone app	6 months	none	6 months	Smoking rates, physical activity, alcohol or fruit and vegetable consumption. Biochemical measures: A hair sample was taken to assess alcohol consumption, cigarette smoking, and recreational drug use.
Partridge et al. (2015)	Two-arm, parallel, RCT	Medicare locals; 18 to 35 year olds with a BMI of 23.0 to 24.9 kg/m^2^	241 (I = 110, C = 104); 28.1, 27.2	8 text messages and 1 email weekly, 5 personalized coaching calls, a diet booklet, and access to resources and mobile phone apps on a website. >TXT2BFiT	Mobile phone apps on a website	12 weeks	4 text messages and printed dietary and physical activity guidelines	12 weeks	Self-reported weight and dietary intake of vegetables, sugary soft drinks, energy-dense takeout meals and physical activity (MET-minutes).
Peyman et al. (2018)	Quasi	Women visitors in health centers; older than 18 years and overweight.	360 (I = 180, C = 180); 31.9, 33.4.	Educational website related to physical activity including film, video, video clips, educational material, CD, self-monitoring program, text messages daily	Web-based media, mobile phone.	2 months	None	6 months	IPAQ, mean score of knowledge, attitude, level of physical activity, MET-minutes/week, BMI.

Note: I, intervention group; C, control group; IPAQ, International Physical Activity Questionnaire; MET, metabolic equivalents; BMI, body mass index; RCT, randomized controlled trial; SSBs, sugar-sweetened beverages.

**Table 2:** Theoretical basis and measurement of physical activity (PA) in the developed Intervention.

First Authors (Published Year)	Study Design	Health Behavior Change Theory	Measurement of PA	Tool	Analysis
Allman-Farinelli et al. (2016)	Two-arm, parallel, RCT	Transtheoretical model	IPAQ (MET-minutes)	Questionnaire	Change in frequency (days) and minutes
Hebden et al. (2014)	Two-arm, parallel, RCT	Transtheoretical model	IPAQ (MET-minutes), daily step counts	Questionnaire, actigraph accelerometer	MVPA, light PA, total PA (minutes per week, MET-minutes /week), sitting time, sedentary time. Vigorous (>5738 counts per minute), moderate (1952–5737 counts per minute), light (101–1951 counts per minute) and sedentary (≤100 counts per minute) activities
Epton et al. (2014)	Two-arm, RCT	Theory of Planned Behaviors, self-efficacy	IPAQ-SF	Questionnaire in the past 7 days	Total MET and sitting mean hours
Partridge et al. (2015)	Two-arm, parallel, RCT	Transtheoretical model	IPAQ (MET-minutes)		Vigorous PA (MET-minutes per week, days per week), walking, moderate PA, total PA
Peyman et al. (2018)	Quasi	None	IPAQ	Questionnaire	Total MET-minutes/week.

Note: IPAQ, International Physical Activity Questionnaire; MET, metabolic equivalents; MVPA, moderate and vigorous physical activity; PA, physical activity; SF, short form.
